# Randomized pilot trial for the efficacy of the MMF07 foot massager and heat therapy for restless legs syndrome

**DOI:** 10.1371/journal.pone.0230951

**Published:** 2020-04-02

**Authors:** Ariane Park, Katherine Ambrogi, Erinn M. Hade

**Affiliations:** 1 Department of Neurology, Madden Center for Parkinson’s Disease and Related Disorders, The Ohio State University Wexner Medical Center, Columbus, Ohio, United States of America; 2 Department of Biomedical Informatics, Center for Biostatistics, The Ohio State University Wexner Medical Center, Columbus, Ohio, United States of America; IRCCS E. Medea, ITALY

## Abstract

**Background:**

Restless Legs Syndrome (RLS) is a sensorimotor condition with a wide range of severity. Symptoms negatively affect sleep and quality of life. Pharmacologic options are not universally effective and side effects are common. Objective data regarding non-pharmacologic treatment is limited. The study objective was to evaluate the efficacy of the MMF07 foot massager and heat therapy on the severity of RLS symptoms.

**Methods:**

In this pilot randomized controlled trial, twenty-eight patients with diagnosed, bothersome RLS were randomized to four treatment arms: no active intervention (n = 7), foot massager (n = 8), heat therapy (n = 6), and foot massager plus heat therapy (n = 7). Participants completed the RLS Severity Scale, RLS Quality of Life questionnaire, and the Medical Outcomes Study Sleep scale at the baseline visit and at the 4-week follow up visit.

**Results:**

Four weeks post randomization, participants in the massager group had significant improvement in the RLS severity score (average difference: -9.0, 95% CI: -16.3, -1.7, p = 0.017) and sleep scale (average difference: -22.0, 95% CI: -36.5, -7.5, p = 0.005) compared to the no intervention group. The heat alone group had a significant improvement in the sleep scale compared to the no-intervention group (average difference: -17.4, 95% CI: -32.5, -2.3, p = 0.026). Quality of life improved in the massage only group compared to control (average difference 25.3, 95% CI: -2.4, 53.0, p = 0.072).

**Conclusions:**

Results suggest that the MMF07 foot massage device and heat therapy may be feasible and effective treatment options to improve RSL symptoms.

## Introduction

Restless legs syndrome (RLS) is characterized by abnormal, uncomfortable sensations, typically present in the legs and sometimes in the arms, which are temporarily relieved by movement. RLS symptoms have a circadian pattern, with symptoms worsening during in the evening hours. These clinical symptoms are used in the clinical diagnosis of RLS [1].

RLS symptoms can lead to significant sleep loss and disruption in quality of life. In one large population study, 88% of patients with RLS reported sensory problems and pain, and 76% reported sleep disturbances [2]. In fact, RLS patients have reported similar disruption in quality of life as those with other chronic disorders such as type 2 diabetes mellitus, osteoarthritis, depression and hypertension [2,3]. It may be that decreased daytime alertness and emotional distress may be secondary to the sleep disturbances due to RLS [4].

RLS treatment is mainly pharmacologic, but these interventions have limitations. Dopaminergic agonists are considered the gold standard treatment for RLS [5]. However, dopaminergic medications are associated with “augmentation,” a phenomenon characterized by RLS symptoms that start earlier in the daytime, and ascend up the body, sometimes involving the arms and trunk [6]. Also, dopaminergic therapies are associated with impulse control disorders, such as punding, pathologic gambling, binge eating and hypersexuality [7]. Other treatments include anti-epileptics, benzodiazepines and opiates but these can cause sedation, dizziness and mood changes [8]. There is limited data supporting the use of clonidine and buproprion, and oral iron has been determined to not be efficacious in iron-sufficient patients [9]. Its benefit for patients with low peripheral iron is unclear [10].

Given the limitations of current pharmacologic interventions for RLS and in light of the severity of RLS symptoms on quality of life, effective non-pharmacologic, non-invasive treatments would be an important advancement in the treatment of this aggravating disease. However, few quality randomized controlled clinical trials have been conducted [11]. Non-pharmacologic therapies that have been evaluated for the treatment of RLS include enhanced external counterpulsation (EECP) [12], sclerotherapy [13], deep brain stimulation therapy [14], and acupuncture [15]. Non-invasive treatments that have been recommended for RLS patients include warm or cool baths, massage, exercise, and staying mentally active as symptoms can be triggered when patients are bored. To our knowledge, there have been no clinical trials evaluating the efficacy of heat therapy in treating RLS symptoms, although one study suggested that electrical external sensory stimuli may lead to less leg discomfort in RLS [16]. In May 2014, the Food and Drug Administration (FDA) granted commercial clearance for a vibrating pad, Relaxis®, to be marketed for improvement in the quality of sleep for patients with primary RLS. A four week study concluded that treatment with such vibrating pads safely improved sleep in patients with RLS [17]. A 2018 review of 11 randomized controlled trials studies comparing non-pharmacological interventions for restless legs syndrome to alternative or no treatment controls concluded that repetitive transcranial magnetic stimulation, exercise, compression devices, counterstrain manipulation, infrared therapy and acupuncture, cryotherapy and yoga may improve RLS severity and sleep in patients with RLS [11].

Given the relative lack of controlled clinical trials evaluating non-pharmacologic treatments for RLS, we conducted a randomized pilot trial examining whether a foot massage device and/or heating therapy improved severity of restless legs symptoms as measured by the International Restless Legs Severity Scale. Secondary outcomes included quality of life and sleep as measured by the Restless Legs Quality of Life Questionnaire and Medical Outcomes Sleep Study scale. We hypothesized that participants in the active treatment arms would experience attenuation of RLS symptoms and improvement in quality of life and sleep.

## Methods

### Study design

This was a prospective pilot clinical trial using a 2 x 2 factorial design, to examine the improvement of RLS symptoms with use of the MMF07 Foot massager with or without heat therapy after 4 weeks of therapy. Eligible patients with restless legs syndrome (RLS) were randomized to one of four treatment arms on a rolling basis: no intervention, foot massage device alone, heat therapy alone, and foot massage device plus heat therapy. Participants were recruited from our movement disorders clinic, and via informational letters, fliers and post cards sent to local sleep medicine, primary care and internal medicine clinics. Several participants contacted us after finding the study listed on the clinicaltrials.gov website. To balance severity of symptoms across treatment cohorts, the randomization scheme was stratified by mild/moderate versus severe/very severe RLS symptoms as measured by the International Restless Legs Severity Scale (IRLSS), a 40 point scale measuring severity of restless legs symptoms [18]. Patients were asked to complete this at the initial visit prior to randomization.

This study was approved by the Biomedical Institutional Review Board at The Ohio State University Wexner Medical Center (study number 2015H0107). Written informed consent was obtained from all subjects. We confirm that we have read the Journal’s position on issues involved in ethical publication and affirm that this work is consistent with those guidelines. The principles outlined in the “Declaration of Helsinki” were followed. The trail was registered as ClinicalTrials.gov Protocol Record NCT02526277.

#### Enrollment

A partial waiver of consent allowed us to assess eligibility prior to formal consent. After participant eligibility was determined, participants came in for the initial visit, where the International Restless Legs Syndrome Study Group diagnostic criteria was reviewed and confirmed [1], study protocol was explained and informed consent documents were signed, prior to randomization. The study research coordinator randomized eligible participants to one of the four treatment groups. The principle investigator was blinded to treatment assignments.

#### Randomization

The randomization scheme was stratified by RLS severity as assessed by the IRLSS score (mild/moderate and severe/very severe) and permuted blocks of varying sizes allocated participants to one of the four treatment groups. The randomization scheme was generated by the study statistician, and was not available to the clinical team until the time of randomization. Random allocation to treatment group and study data collection and tracking was implemented in REDCap, an electronic data capture tool hosted at The Ohio State University Wexner Medical Center [19].

#### Inclusion and exclusion criteria

Participants were required to be between the ages of 18–75 at the time of randomization and be diagnosed with restless legs syndrome according to the diagnostic criteria of the International Restless Legs Syndrome Study Group [1]. In addition, potential participants had to report: 1) bothersome RLS symptoms, despite best medical therapy, 2) that they were stable on all RLS medication for at least 4 weeks prior to enrollment, 3) were able to read and write in English to be able to complete home diary cards and questionnaires, 4) all women of childbearing age had to be using an acceptable form of birth control.

Participants were excluded if: 1) RLS was secondary associated with end stage renal disease, iron deficiency or pregnancy, 2) had been formally diagnosed with a concomitant sleep disorder including insomnia or obstructive sleep apnea, 3) had insufficient vision to be compliant with study procedures, 4) had been diagnosed with any other condition (other than the primary indications), which in the opinion of the investigators might contribute to difficulty complying with the protocol.

#### Intervention groups

1) No intervention: Participants randomized to the ‘no intervention/usual practice’ group were asked to not alter their nighttime routine.

2) Massager: Participants randomized to use the foot massager (the massager alone or in combination with heat therapy) were instructed to place the device on a hard surface, plug the unit into a wall outlet and turn the unit to the speed of 3. They were then instructed to place their feet (bare, with sock or shoes) onto the footpad. Then participants were able to adjust the speed to a desired level of comfort. After 30 minutes of use at bedtime, participants were asked to turn the massager off and unplug the device. Participants were provided with a home diary to record duration of use and device setting on a nightly basis. They were asked to otherwise not alter their nighttime routine.

The MMF07 Foot Massager is manufactured by Medmassager. It is engineered in an International Organization for Standardization (ISO) 9001–2008 manufacturing facility and is Canadian Standards Association (CSA) certified for safety and reliability. The massager is FDA certified for therapeutic use under regulation number: 890.5660.

This massager utilizes a motion footplate to move the feet and legs in a circle 20 microns in diameter, creating a vibrating sensation. The unit has an adjustable variable controller, allowing for more or less stimulation. The unit is constructed of a plastic shell, footplate frame and a hypoallergenic footpad surface. The motor is mounted to a metal sub frame and attached to the footplate frame with a 20 micron bearing and insulation cap. The unit has never been tested clinically before. The only adverse effect that has been reported by users to the manufacturer is an itchy sensation in the feet and/or legs during use.

3) Heat Therapy: Participants randomized to use heat therapy (either the heat therapy alone or the massager and heat therapy group) were given an electric Sunbeam Heat Pad with UltraHeat Technology. The UltraHeat Technology maintains a consistent heat level at low, medium, or high settings. Participants were instructed to keep the cloth cover on the heating pad at all times. They were to place the pad across their thighs, and start at the medium heat setting. Participants were then able to adjust the heat setting to their desired level of comfort. After 30 minutes of use at bedtime, they were to turn the pad off and unplug the unit. Participants were provided with a home diary to record duration of use and pad setting on a nightly basis. Patients were asked to otherwise not alter their nighttime routine.

4) Combined massager and heat therapy: Participants who were randomized to use the combined massager and heat therapy were advised to use the two interventions simultaneously, each as described above, for 30 minutes total before bedtime.

### Outcome measures

At four weeks post randomization, participants completed self-administered outcome measures. All participants completed these in person, with the exception of one participant who completed the outcome measures via email due to the distance to our site and personal health issues making travel difficult.

#### Primary outcome

The primary outcome measure was The International Restless Legs Severity Scale(IRLSS) [18]. The IRLSS is a 40-point scale measuring severity of restless legs symptoms; this scale was collected at baseline and was utilized as strata to balance severity in randomized groups.

#### Secondary outcomes

Secondary outcomes included the Restless Legs Quality of Life Questionnaire [20], a series of 18 questions that are scored such that lower scores indicate worse quality of life, and the Medical Outcomes Sleep Study scale [21], a series of 12 questions assessing quality of sleep, with values ranging from 1 to 6, and an additional dichotomous indicator of optimal sleep quality.

### Other participant data collection

Demographics, medical history, and information about alcohol, drug and tobacco use was collected at the baseline visit. Medications and vital signs were recorded at each visit. Urine pregnancy tests were performed on one female participant who was of child bearing potential at baseline, prior to randomization, and again at week 4.

Adverse events were recorded at week 4 or as reported by the patients. Home diaries were collected at the week 4 visit. Home diaries recorded the duration of each intervention used in minutes, as well as massage and heat settings when applicable, for each day a participant was on therapy.

A unique identifier was generated for study-related patient data (case report forms). Study-related documents were kept in a locked cabinet in a locked office. A separate list (paper-only) was made containing the unique identifier and the name of each participant. This list was kept separate from the study related documents. The study REDCap database is stored on the OSUWMC secured network and is only accessible by authorized study investigators and staff. Paper files are secured in a locked office and kept for 5 years.

### Statistical considerations

#### Design

The original sample size estimated for at least 80% power for the comparison of all 3 active treatments to control was 10 per group (40 total), with an adjusted one-sided type-one error rate of 10% (adjusted for 3 primary comparisons). These calculations assumed an effect size of a 5-point difference with the control group in the International Restless Legs Severity Scale, for the main effects of the foot massage device and heat therapy, and for their combined effect, assuming a standard deviation of 4 within each cohort. Given challenges to recruitment, the trial team halted enrollment at 28 patients, where it was expected based on the original design characteristics that power for the planned design effect (5-point difference between each treatment and control), specified type-one error rate, and a standard deviation of 4 in the scale, was expected to be over 70%, and over 80% for the same treatment effect size with smaller variability (sd = 3.5).

### Statistical analysis

Demographic and baseline characteristics are summarized overall and by treatment group. Linear regression models were used to test and to estimate the primary effects of interest: the comparison of the foot massage device alone, heating therapy alone and the combination of the massager and heating therapy versus no therapy for the IRLSS, and for all continuous secondary outcomes. Modified Poisson regression was used to estimate the proportion of patients reaching optimal sleep, a dichotomous outcome, as well as the associated risk differences between treatment groups. Exploratory analysis assessed the impact of baseline adjustment of each measure, for continuous outcomes by linear regression. All data management and analyses were conducted in Stata version 15.0 [22]. Confidence intervals and p-values are all two-sided and at the nominal level.

## Results

**Fig 1:**
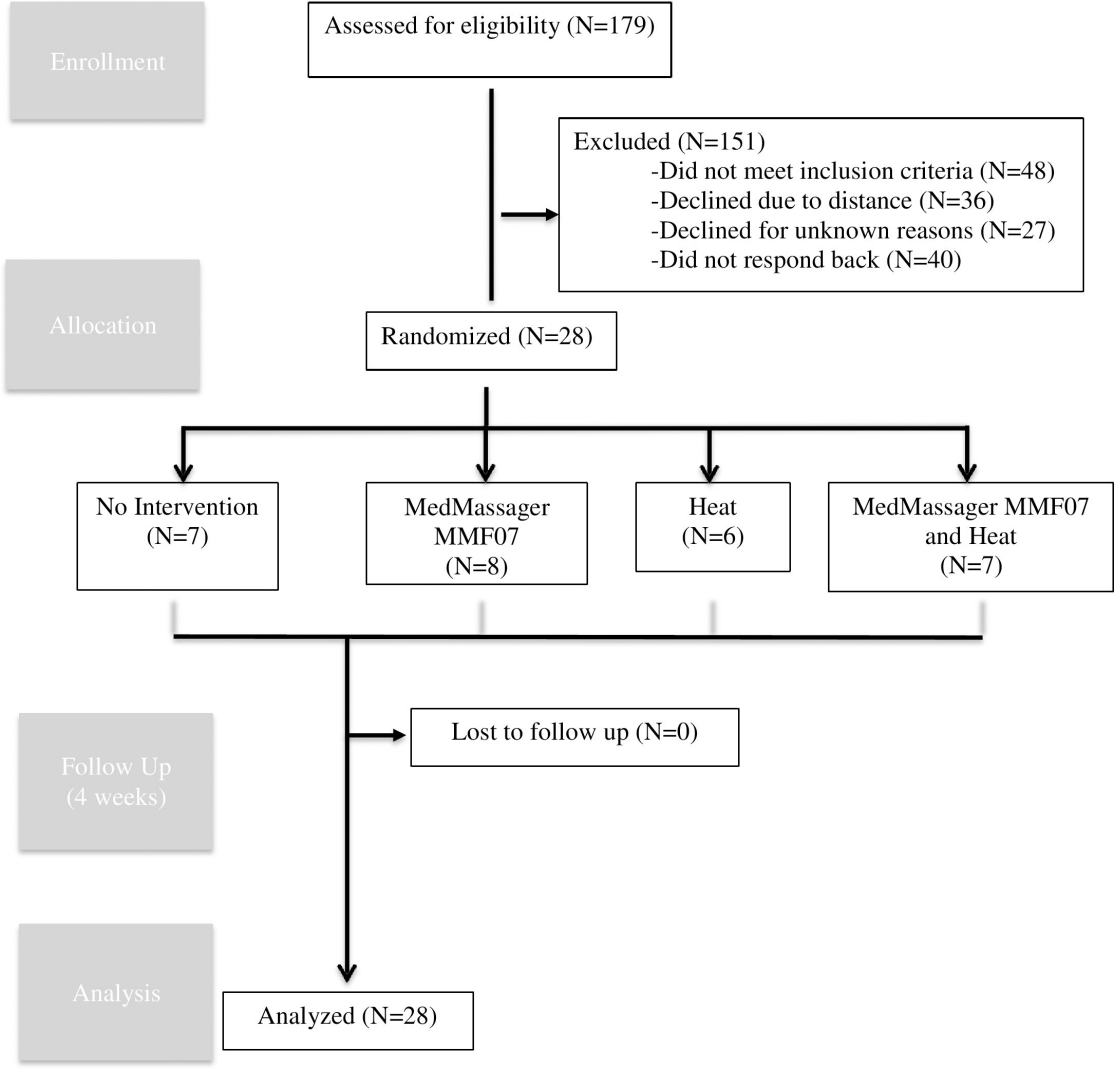
Flow of participants at each stage of the trial.

Between January 2016 and June 2018, 28 participants between the ages of 18–75 diagnosed restless legs syndrome according to the diagnostic criteria of the International Restless Legs Syndrome Study Group (1) were registered and randomized. Seven patients were randomized to no intervention, eight to the foot massage device, six to heat therapy, and seven to the foot massage device plus heat therapy (Fig 1). All participants completed the primary outcome assessment either in person (n = 27) or via email (n = 1).

Demographics and clinical characteristics of the patients are outlined in Table 1. Groups were generally balanced in terms of age, race, ethnicity and gender. Baseline RLS severity (mild/moderate versus severe/very severe) was balanced across the four treatment arms by design. 26 out of the 28 participants were on oral medication to treat RLS symptoms.

**Table 1:** Patient baseline characteristics.

	Control *n = 7*	Massager Only *n = 8*	Heat Only *n = 6*	Massager and Heat *n = 7*	Total N = 28
*Age (years)*					
*mean(sd)*	54.2 (10.4)	61.1 (13.3)	61.8 (6.2)	58.4 (12.0)	58.8 (10.8)
*median (min*, *max)*	52.7 (39.2, 70.9)	65.8 (36.5, 73.1)	59.6 (55.6, 72.8)	59.9 (34.9, 72.4)	59.6 (34.9, 73.1)
*White race*, *n (%)*	7 (100)	8 (100)	6 (100)	7 (100)	28 (100%)
*Non-Hispanic ethnicity*, *n (%)*	7 (100)	8 (100)	6 (100)	7 (100)	28 (100%)
*Female gender*, *n (%)*	4 (57.1)	7 (87.5)	3 (50.0)	5 (71.4)	19 (67.9%)
*Ever Smoker*, *n (%)*	3 (42.9)	1 (12.5)	0 (0)	2 (28.6)	6 (21.4%)
*Married*	3 (42.9)	3 (37.5)	0 (0)	2 (28.6)	20 (71.4%)
*RLS Severity*					
*Mild/Moderate*	3 (43.9)	4 (50)	3 (50)	4 (57.1)	14 (50%)
*Severe/Very severe*	4 (57.1)	4 (50)	3 (50)	3 (43.9)	14 (50%)

Table 2 summarizes the primary and secondary outcomes by treatment group, and provides the estimated contrasts between each active treatment and the control condition of no active intervention. After four weeks of follow up, there was evidence that the participants in the foot massager only group had improved (lower) RLS severity scores. RLS severity score was on average 12.3 (sd = 4.2) in the massage only group, compared to 21.3 (sd = 8.5) in the no intervention group, a decrease of 9 points (average difference: -9.0, 95% CI: -16.3, -1.7, p-value = 0.017). These substantial decreases in RLS severity scale score were not observed in the heat therapy group alone (average difference: -3.6, 95% CI: -11.8, 3.9) or the combined foot massager and heat therapy group (average difference: -4.4, 95% CI: -12.0, 3.1) compared to the no intervention group. Table 2 also includes estimates of these differences with the control group, adjusted for baseline severity group which was used to stratify the randomization scheme. Results and inferences remain consistent. Given the variation in RLS severity score at baseline, we explored the estimated effect of treatment compared to no treatment, after adjustment for baseline score. Similarly, there was strong evidence that the average change from baseline in the massager only group, as compared to the no-intervention group, was improved (average change from baseline compared to control: -6.3, 95% CI: -12.0, -0.54, p-value = 0.033).

**Table 2:** Comparison of outcomes between treatment groups.

	Control *mean (sd) n = 7*	Massager Only *mean (sd) n = 8*	Heat Only *mean (sd) n = 6*	Massager and Heat *mean (sd) n = 7*
*Restless Leg Syndrome Severity Scale*				
*Baseline*	24.6 (8.1)	19.9 (9.8)	22.8 (6.6)	19.9 (5.1)
*4 week follow up*	21.3 (8.5)	12.3 (4.2)	17.3 (8.1)	16.9 (6.2)
*Difference with Control* (95% CI) p-value	-	-9.0 (-16.3, -1.7) 0.017	-3.6 (-11.8, 3.9) 0.309	-4.4 (-12.0, 3.1) 0.238
*Adjusted difference with Control* (95% CI)^^^ p-value		-8.4 (-14.1, -2.7) 0.005	-3.4 (-9.5, 2.8) 0.268	-3.2 (-9.2, 2.7) 0.267
*Average change score compared to Control* (95% CI)* p-value	-	-6.3 (-12.0, -0.5) 0.033	-2.9 (-9.0, 3.1) 0.324	-1.7 (-7.6, 4.3) 0.566
*Restless Legs QOL summary score*				
*Baseline*	49.8 (26.2)	59.1 (40.2)	62.6 (20.0)	68.0 (14.5)
*4 week follow up*	57.1 (32.6)	82.4 (20.9)	66.7 (25.6)	74.2 (19.1)
*Difference with Control* (95% CI) p-value	-	25.3 (-2.4, 53.0) 0.072	9.5 (-19.3, 38.4) 0.502	17.0 (-10.7, 44.8) 0.216
*Adjusted difference with Control* (95% CI)^^^ p-value		21.4 (-1.9, 44.7) 0.070	7.6 (-16.6, 31.8) 0.521	13.2 (-10.1, 36.5) 0.254
*Average change score compared to Control* (95% CI)* p-value	-	13.0 (-8.0, 34.0) 0.215	0.6 (-20.9, 22.1) 0.956	4.4 (-16.7, 25.5) 0.671
*Medical Outcomes sleep scale*: *Sleep Problems Index 9 item*				
*Baseline*	52.6 (14.1)	46.7 (19.9)	43.6 (19.2)	39.8 (9.3)
*4 week follow up*	48.6 (18.4)	26.6 (9.9)	31.2 (15.8)	37.0 (3.7)
*Difference with Control* (95% CI) p-value	-	-22.0 (-36.5, -7.5) 0.005	-17.4 (-32.5, -2.3) 0.026	-11.6 (-26.1, 2.9) 0.112
*Adjusted difference with Control* (95% CI)^^^ p-value		-21.4 (-36.1, -6.6) 0.006	-17.1 (-32.3, -1.8) 0.030	-11.0 (-25.7, 3.7) 0.136
*Average change score compared to Control* (95% CI)* p-value	-	-15.7 (-27.2, -4.1) 0.010	-12.1 (-23.9, -0.2) 0.046	-4.0 (-15.7, 7.7) 0.486
*Optimal Sleep Indicator*				
*Baseline*, *n(%)*	2 (28.6%)	3 (37.5%)	1 (16.7%)	4 (57.1%)
*4 week follow up*, *n(%)*	2 (28.6%)	4 (50.0%)	1 (16.7%)	4 (57.1%)
*Difference in proportions with Control* (95% CI) p-value	-	21.4% (-27.6%, 70.5%) 0.392	-11.9% (-57.6%, 33.7%) 0.609	28.6% (-22.0%, 79.1%) 0.268
*Adjusted difference in proportions with Control* (95% CI)^^^ p-value		19.5%(-29.7%, 68.8%) 0.437	-13.8% (-54.7%, 27.1%) 0.508	23.3% (-22.1%, 68.7%) 0.314

*^* Adjusted estimates account for stratification by baseline IRLSS group (mild/moderate vs. severe/very severe).

* The treatment effect is the estimated average difference between each active treatment group and control group adjusted for the baseline measure (RLS, RLQOL, MOSS). These estimates were obtained through linear (RLS, QOL, Sleep Index) or modified Poisson regression models (Optimal Sleep Indicator).

All p-values are two-sided.

The most dramatic impact on secondary outcome measures was observed for the Medical Outcome sleep scale. There was evidence in favor of lower scores on the 9-item sleep problems scale for the massager alone group compared to the no intervention group (average difference: -22.0, 95% CI: -36.5, -7.5, p-value = 0.005). Moreover, there was also evidence that the heat alone group improved on the sleep scale, with an average decrease as compared to no-intervention of -17.4 (95% CI: -32.5, -2.3, p-value: 0.026). The evidence was less strong for the group provided with both intervention modalities, average difference with the no-intervention group: -11.6, 95% CI: -26.1, 2.9, p-value = 0.11. These patterns were similar for the estimated average change scores, as compared to no-intervention change.

Participants in all treatment arms reported improvement in quality of life as measured by the RLS Quality of Life Questionnaire (QOL). The estimated difference in average QOL between the massage only group and the no intervention group was 25.3 points with a 95% CI: -2.4, 53.0, p-value = 0.072. Differences between the heat alone and the heat + massage group and the no-intervention group were not as strong.

Both the number of days used and the average length of time used each day was near the recommended target for each therapeutic modality (Table 3). Participants randomized to the massager alone used the device on average for 24.8 minutes (95% CI: 12.6, 36.9) and for 22.3 minutes (95% CI: 10.1, 34.4) in the massager + heat group. The average duration of heat use was slightly higher, with 30.8 minutes on average in the heat alone group (95% CI: 29.5, 32.1) and 31.3 minutes on average (95% CI: 30.1, 32.5) in the combination group.

**Table 3:** Compliance with therapy in treatment groups.

	Control *mean (sd) n = 7*	Massager Only *mean (sd) n = 8*	Heat Only *mean (sd) n = 6*	Massager and Heat *mean (sd) n = 7*
*Duration of use (minutes per day used)*				
*Massager at 4 week follow up (95% CI)*	-	24.8 (12.6, 36.9)		22.3 (10.1, 34.4)
*Heat at 4 week follow up (95% CI)*	-		30.8 (29.5, 32.1)	31.3 (30.1, 32.5)
*Days of use during 4 week follow up*				
*Massager (95% CI)*	-	24.5 (18.7, 30.3)		28.1 (21.9, 34.4)
*Heat (95% CI)*	-		28.7 (27.4, 29.9)	28.1 (27.0, 29.3)

Adverse events were rare and those potentially related to the massager were mild. These included: worsening neuropathy, tingling in the feet, intermittent itching of feet and ankles, and leg jerks. The more severe adverse events of flu (moderate), sciatica (moderate), and migraine (severe) were unrelated to the study treatments.

## Discussion

Safe and effective non-pharmacologic treatments are needed for RLS. This randomized prospective pilot study demonstrates that participants using a foot massage device experienced improvement in both the primary and secondary outcome measures. RLS severity scores, the primary study outcome measure, improved the greatest in those using the massager compared to those in the other groups. Participants using the massager also reported the greatest improvement in secondary outcome measures: Medical Outcomes of Sleep and RLS QOL scales compared to the other groups.

There are few published studies on use of massage/vibration to compare or contrast with the outcomes of the current study. A 2013 study evaluating pooled data from two randomized, double-blind, prospective clinical trials found improved sleep in patients receiving 4 weeks of treatment with a vibrating pad compared to shams, but failed to show significant improvements in RLS severity or quality of life [17]. However, the pads used in the other studies were of a different design than the massage/vibration device used in the current study.

RLS is genetically heterogeneous with wide phenotype variability. Moreover, it remains debated to what degree RLS is a central versus peripheral nervous system disorder. In primary RLS, nerve conduction studies show normal values, however studies have suggested that RLS patients may have abnormal temperature perception, possibly due to small fiber neuropathy [23] or impairment in central somatosensory processing [24]. The argument against a purely peripheral etiology for RLS includes the fact that patients with amputations have developed RLS symptoms responsive to dopamine agonists [25][26]. While some have suggested a correlation with microvascular abnormalities [27] and peripheral hypoxia [28], there is sufficient evidence that the dopaminergic system and brain iron metabolism play a significant role in the pathophysiology of RLS [29][30].

The pathways and circuitry that contribute to RLS symptomatology are poorly understood making it difficult to hypothesize the mechanism that might underlie the effect of a vibratory stimulus on decreasing RLS symptoms. fMRI studies have shown involvement of the red nucleus and brainstem areas during limb movements associated with RLS, and activation of cerebellar and thalamic regions during sensory symptoms [31]. It has been suggested that interactions between sensory cortical regions play a role in vibratory analgesia [32], and this may explain the vibratory benefit on RLS symptoms. However, it has also been demonstrated that there is a circadian variation at the level of spinal cord activity leading to a nocturnal increase in spinal cord excitability as demonstrated by increased lower extremity flexor withdrawal reflexes and crossed extensor reflexes in individuals with RLS compared to controls [33]. We speculate that there may be a diurnal sensitization of dorsal horn central afferent pathways in RLS individuals. One might postulate that the vibratory stimulus of the foot massager might desensitize the dorsal columns (vibratory sensory pathways) and thereby change the balance of activity in the cord resulting in relief of nocturnal symptoms.

Interestingly, though RLS participants using heat therapy alone did not report as dramatic improvement in RLS severity scores, they were also noted to have benefit in sleep scores. Thermotherapy is known to have an analgesic effect, as it increases blood flow and connective tissue extensibility [34], and is effective in treating musculoskeletal conditions [35]. However, thermotherapy has only been anecdotally beneficial and has not been objectively studied in RLS. Heat likely affects a different combination of peripheral and central factors contributing to RLS symptoms compared to vibratory stimulation. The fact that simultaneous administration of heat and massage treatment was not additive, but rather less than massage alone suggests that these interactions need to be considered.

Limitations of this study include its small sample size and subsequent limited power to detect effects of clinical interest. A relatively short follow up period of 4 weeks was chosen to encourage compliance, and reassuringly there were no inconsistencies regarding duration of use of each therapeutic modality during this time period. However, this short intervention period may have not been enough time to elicit other significant changes. Furthermore, RLS is a subjective condition without objective biomarkers. Since the dopaminergic system seems to be involved the pathophysiology of this condition, there may be inherent issues with placebo response. Given the open design of this study where participants were aware of their treatment modality, we recognize that the observed improvements reported by patients may be in part due to those patients’ belief in the benefit of the treatments, rather than the treatments themselves. We did have a control arm, however future work may evaluate this potential placebo effect with a placebo-controlled group. Silva et al. found that placebo responses were greater in clinical trials lasting longer than 12 weeks, those evaluating pharmacologic interventions, and in trials involving idiopathic RLS rather than secondary RLS [36]. Larger trials of longer duration are warranted to further investigate the clinical meaningfulness of vibratory stimuli and heat in the treatment of RLS as these non-pharmacologic interventions may be effective, safe, and economical treatments options.

## Supporting information

S1 Checklist (DOC)

S1 Data (DOCX)
