# Supplementary material for: Randomized pilot trial for the efficacy of the MMF07 foot massager and heat therapy for restless legs syndrome
Source: PLoS One. 2020 Apr 2;15(4):e0230951. doi: 10.1371/journal.pone.0230951 (PMC7117678; doi:10.1371/journal.pone.0230951)
Supplement: S1 Data — (DOCX) [file pone.0230951.s002.docx]

**Research Protocol**

A randomized prospective study on the efficacy of the MMF07 Foot Massager and heat therapy treatments for Restless Legs Syndrome

**Principal Investigator:**

Ariane Park, MD, MPH

Assistant Professor

Department of Neurology

395 W. 12^th^ Ave., 6^th^ Floor

Columbus, OH 43210

Email: [Ariane.Park@osumc.edu](mailto:Ariane.Park@osumc.edu)

Phone: 614-293-4969

Fax: 614-293-6111

**Co-Investigators:**

Kathleen Moore CNP

Nurse Practitioner

Department of Neurology

395 W. 12^th^ Ave., 7th Floor

Columbus, OH 43210

Email: [kathleen.moore@osumc.edu](mailto:Andrea.Malone@osumc.edu)

Phone: 614-293-4969

Fax: 614-293-6111

**Key Personnel:**

Katherine Ambrogi RN

Clinical Research Coordinator

Neurology

2006 Kenny Road

Columbus, OH 43210

Email: [katherine.ambrogi@osumc.edu](mailto:Paige.Pancake@osumc.edu)

Phone: 614-688-6685

**Statistician:**

Allison M. Wehr, MS

Biostatistician

Center for Biostatistics

2012 Kenny Road

Columbus, OH 43221

Email: [allison.wehr@osumc.edu](mailto:allison.wehr@osumc.edu)

Phone: 614-293-2999

**I. Study Objectives**

We propose to conduct a prospective clinical trial using a 2 x 2 factorial design; patients with restless legs syndrome (RLS) will be randomized to one of four treatment arms: no intervention, MMF07 Foot Massager device alone, heating therapy alone, and MMF07 Foot Massager device plus heating therapy.

Few clinical trials have looked into non-pharmacologic, non-invasive treatments for restless legs syndrome, despite reports that massage, baths and vibration can alleviate RLS symptoms. We would like to assess whether the MMF07 Foot Massager device and/or heating therapy is associated with improved severity of restless legs symptoms as measured by the International Restless Legs Severity Scale. Secondary outcomes include evaluating potential changes in both quality of life and sleep as measured by the Restless Legs Quality of Life Questionnaire and Medical Outcomes Sleep Study scale, respectively. Current therapies for RLS are limited, therefore investigating effective non-invasive treatments that attenuate symptoms would be an important advancement in the treatment of this aggravating disease.

**II. Background and Rationale**

Restless legs syndrome (RLS) is characterized by abnormal, uncomfortable sensations, typically present in the legs, which are temporarily relieved by movement. The pathophysiology of RLS is unknown. Several theories exist, including microvascular abnormalities ([1](#_ENREF_1)), peripheral hypoxia ([2](#_ENREF_2)) and changes in dopaminergic pathways which may lead to lower iron levels in the brain ([3](#_ENREF_3)).

Pharmacologic treatments for RLS include dopamine agonists, anti-epileptics, benzodiazepines and opiates. There is limited data supporting the use of clonidine, baclofen, tramadol and magnesium as first- or second-line therapy. Iron supplementation, orally or intravenously, is recommended for patients with low serum ferritin levels. The only randomized, double-blind, placebo-controlled study that evaluated oral iron therapy for RLS did not provide sufficient evidence to show that iron sulfate was a good empiric treatment for RLS ([4](#_ENREF_4)).

There are limitations to pharmacologic therapies. For example, dopaminergic medications, considered the gold standard treatment for RLS, are associated with “augmentation,” a phenomenon characterized by RLS symptoms that start earlier in the daytime, and ascend up the body, sometimes involving the arms and trunk. Also, dopaminergic therapies are associated with impulse control disorders, such as punding, pathologic gambling, binge eating and hypersexuality. Anti-epileptics, benzodiazepines and opiates can cause sedation, dizziness and mood changes.

Non-pharmacologic therapies that have been evaluated for the treatment of RLS include enhanced external counterpulsation (EECP) ([5](#_ENREF_5)), sclerotherapy ([6](#_ENREF_6)), deep brain stimulation therapy ([7](#_ENREF_7)), and acupuncture ([8](#_ENREF_8)). Non-invasive treatments that are recommended for RLS patients include warm or cool baths, massage, exercise, and staying mentally active as symptoms can be triggered when patients are bored. To our knowledge, there have been no clinical trials evaluating the efficacy of heating therapy in treating RLS symptoms. A recent study showed that external sensory input may lead to less leg discomfort in RLS ([9](#_ENREF_9)). In May 2014, the Food and Drug Administration (FDA) granted commercial clearance for the Relaxis Pad to be marketed for improvement in the quality of sleep for patients with primary RLS. This device, developed by Sensory Medical, is a vibrating pad that patients can place at their site of discomfort while remaining in bed. A four week study published in a non-MEDLINE-indexed journal found that treatment with such vibrating pads safely improved sleep in patients with RLS ([10](#_ENREF_10)).

Given this knowledge gap in the realm of non-pharmacologic, non-invasive treatments for RLS, our study aims to explore whether the MMF07 Foot Massager and/or heating therapy may alleviate the symptoms of RLS.

**III. Procedures**

*A. Research and Design*

**Subject Selection/Sample**

- Inclusion criteria:

1. Signed an informed consent (ICF), indicating that they have been informed of the procedures to be followed, the experimental nature of the therapy, alternatives, potential benefits, side-effects, risks and discomforts.
2. Patients between 18-75 years old diagnosed with RLS according to the diagnostic criteria of the International Restless Legs Syndrome Study Group ([11](#_ENREF_11)).
3. Subjects should have bothersome RLS symptoms, despite best medical therapy
4. Subjects should be stable on all RLS medication for at least 4 weeks prior to enrollment
5. All subjects must be able to read and write in English in order to be able to complete home diary cards and questionnaires.
6. All women of childbearing age must be using an acceptable form of birth control, including abstinence, IUD or intrauterine system in place for at least 3 months prior to screening, subject or partner using barrier method (e.g., condom, diaphragm, or cervical cap) with spermicide from screening through study completion; partner has a documented vasectomy > 6 months prior to Baseline, Stable hormonal contraception (with approved oral, transdermal, or depot regimen) for at least 3 months prior to screening.

- Exclusion criteria:

1. RLS secondary associated with end stage renal disease, iron deficiency or pregnancy
2. Concomittant sleep disorders
3. Does not have sufficient vision to be complaint with study procedures.
4. Any other condition (other than the primary indications), which in the opinion of the investigators might contribute to difficulty complying with the protocol

**No Intervention**

Patients receiving no intervention will be asked to not alter their nighttime routine.

**MMF07 Foot Massager**

The MMF07 Foot Massager is manufactured by Medmassager, a company located in Arden, NC. The MedMassager brand has been on the market for five years, but versions of this type of massager have been sold in the United States for almost 30 years. The MMF07 is engineered in an International Organization for Standardization (ISO) 9001-2008 manufacturing facility and is Canadian Standards Association (CSA) certified for safety and reliability. The massager is FDA certified for therapeutic use under regulation number: 890.5660.

The MedMassager MMF07 utilizes a circular motion footplate to stimulate circulation in the lower extremities.  Rather than an up and down, vibrating motion which could cause too much stress, the MMF07 moves the feet and legs in a circle 20 microns in diameter. The unit has a variable controller that can be manually adjusted, allowing for more or less stimulation.

The unit is constructed of an ABS plastic shell and footplate frame and a hypoallergenic footpad surface. The motor is mounted to a metal sub frame and attached to the footplate frame with a 20 micron bearing and insulation cap.  The unit has a 10 foot power cord allowing easy access to receptacles without the need for an extension cord.

To operate this device, the power supply cord needs to be inserted into an electrical outlet when the speed switch is in the OFF position. The unit should be turned to the desired speed. When finished, the switch should be turned back to the OFF position, and unit should be unplugged.

The unit has never been tested clinically before. The only adverse effect that has been reported by users to the manufacturer is an itchy sensation in the feet and/or legs during use.

Patients randomized to either the MedMassager MMF07 only or MedMassager MMF07 and heating therapy cohort will be instructed to set this device at setting 3, then increase or decrease the setting to their desired level of comfort. They will be instructed to use this device for thirty minutes at bedtime. Patients will be provided with a home diary to record duration and device setting on a nightly basis. They will be asked to otherwise not alter their nighttime routine.

**Heating Therapy**

Patients randomized to either the heating therapy only or MedMassager MMF07 and heating therapy cohort will be given an electric heating pad, and they will be instructed to use this pad at the medium setting for thirty minutes at bedtime. They can adjust the setting to their level of comfort if needed. They will be provided with a home diary to record duration and pad setting on a nightly basis. Patients will be asked to otherwise not alter their nighttime routine.

*B. Measurement/Instrumentation*

1) International Restless Legs Severity Scale ([12](#_ENREF_12)): a 40 point scale measuring severity of restless leg symptoms. Patients are asked to answer a series of 10 questions each of which have values ranging from 0 to 4 and the points are then added. Higher values are associated with more severe symptoms.

2) Restless Legs Quality of Life Questionnaire ([13](#_ENREF_13)): a series of 18 questions that are scored such that lower scores indicate worse quality of life.

3) Medical Outcomes Sleep Study scale ([14](#_ENREF_14)): a series of 12 questions assessing quality of sleep, with values ranging from 1 to 6

4) Home diary (see attached)

Visits will be at baseline and at week 4. Outcome measures will be self-administered at each visit. Medical history including demographic information and information about alcohol, drug and tobacco use as well as current exercise regimen will be collected at the baseline visit. Medications and vital signs will be recorded at each visit. Urine pregnancy tests will be performed on all females of child bearing potential at baseline prior to randomization and again at week 4. Adverse events will be recorded at week 4 or as reported by the patients. Home diaries will be collected at the week 4 visit.

Anticipated benefits for the subjects in the treatment arms will be attenuation of RLS symptoms and improvement in quality of life and sleep.

**Statistical Considerations:**

*A. Sample Size:*

A total of 40 patients will be accrued to the study with roughly equal allocation to each treatment arm. This sample size will achieve over 90% power to detect a 5 point change in the International Restless Legs Severity Scale for the main effects of heating therapy and MedMassager MMF07 assuming a standard deviation of 4 within each cohort. Additionally we will have roughly 80% power to detect a potential interaction effect between MedMassager MMF07 and heating therapy. Every effort will be made to reduce attrition; consent documents will emphasize the importance of complete data and encourage patients to return for the follow-up visit.

*B. Randomization:*

Patients will be randomized to one of the four treatment options with equal allocation. In an attempt to balance severity across treatment cohorts the randomization scheme will be stratified by baseline severity of RLS symptoms as measured by the International Restless Legs Severity Scale (mild/moderate vs. severe/very severe). The randomization scheme will be generated by the study statistician with varying block sizes and uploaded to a web-based randomization program. After the patients eligibility has been confirmed and consent documents signed the research coordinator will randomize the patient to one of the four treatment options via the web-based randomization program. The PI will not randomize patients or have access to their treatment assignments.

*C. Analysis plan:*

All demographic and baseline characteristics of interest will be summarized both overall and by cohort. Frequencies and percentages will be used to summarize categorical variables and means, standard deviations and other appropriate measures of spread for continuous variables. ANOVA models will be used for testing and to estimate the effects of MedMassager MMF07 alone, heating therapy alone and the potential interaction between MedMassager MMF07 and heating therapy. Sensitivity analyses may be considered to assess the impact of baseline severity and other clinically relevant covariates on the outcomes. We will adjust for multiplicity for all contrasts. Secondary outcomes will be analyzed using a similar approach. Further details will be provided in the Statistical Analysis Plan (SAP).

For study-related patient data (case report forms) a unique identifier will be used. Study-related documents will be kept in a locked cabinet in a locked office. A separate list (paper-only) will be made containing the unique identifier and the name of each participant. This list will be kept separate from the study related documents. Study databases will be stored on a password protected computer network in a locked office and kept for 5 years.

**IV. Bibliography**

*References*

1. Anderson KN, Di Maria C, Allen J. Novel assessment of microvascular changes in idiopathic restless legs syndrome (Willis-Ekbom disease). Journal of sleep research. 2013;22(3):315-21.

2. Salminen AV, Rimpila V, Polo O. Peripheral hypoxia in restless legs syndrome (Willis-Ekbom disease). Neurology. 2014;82(21):1856-61.

3. Dauvilliers Y, Winkelmann J. Restless legs syndrome: update on pathogenesis. Current opinion in pulmonary medicine. 2013;19(6):594-600.

4. Davis BJ, Rajput A, Rajput ML, Aul EA, Eichhorn GR. A randomized, double-blind placebo-controlled trial of iron in restless legs syndrome. European neurology. 2000;43(2):70-5.

5. Rajaram SS, Rudzinskiy P, Walters AS. Enhanced external counter pulsation (EECP) for restless legs syndrome (RLS): preliminary negative results in a parallel double-blind study. Sleep medicine. 2006;7(4):390-1.

6. Kanter AH. The effect of sclerotherapy on restless legs syndrome. Dermatologic surgery : official publication for American Society for Dermatologic Surgery [et al]. 1995;21(4):328-32.

7. Driver-Dunckley E, Evidente VG, Adler CH, Hillman R, Hernandez J, Fletcher G, et al. Restless legs syndrome in Parkinson's disease patients may improve with subthalamic stimulation. Movement disorders : official journal of the Movement Disorder Society. 2006;21(8):1287-9.

8. Hu J. Acupuncture treatment of restless leg syndrome. Journal of traditional Chinese medicine = Chung i tsa chih ying wen pan / sponsored by All-China Association of Traditional Chinese Medicine, Academy of Traditional Chinese Medicine. 2001;21(4):312-6.

9. Rozeman AD, Ottolini T, Grootendorst DC, Vogels OJ, Rijsman RM. Effect of sensory stimuli on restless legs syndrome: a randomized crossover study. Journal of clinical sleep medicine : JCSM : official publication of the American Academy of Sleep Medicine. 2014;10(8):893-6.

10. Burbank F, Buchfuhrer M, Kopjar B. Sleep improvement for restless legs syndrome patients. Part I: pooled analysis of two prospective, double-blind, sham-controlled, multi-center, randomized clinical studies of the effects of vibrating pads on RLS symptoms. Journal of Parkinsonism and Restless Legs Syndrome. 2013:1-10.

11. Allen RP, Picchietti D, Hening WA, Trenkwalder C, Walters AS, Montplaisi J. Restless legs syndrome: diagnostic criteria, special considerations, and epidemiology. A report from the restless legs syndrome diagnosis and epidemiology workshop at the National Institutes of Health. Sleep medicine. 2003;4(2):101-19.

12. Walters AS, LeBrocq C, Dhar A, Hening W, Rosen R, Allen RP, et al. Validation of the International Restless Legs Syndrome Study Group rating scale for restless legs syndrome. Sleep medicine. 2003;4(2):121-32.

13. Abetz L, Vallow SM, Kirsch J, Allen RP, Washburn T, Earley CJ. Validation of the Restless Legs Syndrome Quality of Life questionnaire. Value in health : the journal of the International Society for Pharmacoeconomics and Outcomes Research. 2005;8(2):157-67.

14. Hays RD, Stewart, A.L., editor. Sleep measures. Durham, NC: Duke University Press; 1992.

Name: ________________________________

Start Date: _____________________________

| **Day** | **Duration in minutes** | **Massage setting** |
| --- | --- | --- |
| 1 |  |  |
| 2 |  |  |
| 3 |  |  |
| 4 |  |  |
| 5 |  |  |
| 6 |  |  |
| 7 |  |  |
| 8 |  |  |
| 9 |  |  |
| 10 |  |  |
| 11 |  |  |
| 12 |  |  |
| 13 |  |  |
| 14 |  |  |
| 15 |  |  |
| 16 |  |  |
| 17 |  |  |
| 18 |  |  |
| 19 |  |  |
| 20 |  |  |
| 21 |  |  |
| 22 |  |  |
| 23 |  |  |
| 24 |  |  |
| 25 |  |  |
| 26 |  |  |
| 27 |  |  |
| 28 |  |  |
| 29 |  |  |
| 30 |  |  |
| 31 |  |  |

Name: ________________________________

Start Date: _____________________________

| **Day** | **Duration in minutes** | **Heat setting** |
| --- | --- | --- |
| 1 |  |  |
| 2 |  |  |
| 3 |  |  |
| 4 |  |  |
| 5 |  |  |
| 6 |  |  |
| 7 |  |  |
| 8 |  |  |
| 9 |  |  |
| 10 |  |  |
| 11 |  |  |
| 12 |  |  |
| 13 |  |  |
| 14 |  |  |
| 15 |  |  |
| 16 |  |  |
| 17 |  |  |
| 18 |  |  |
| 19 |  |  |
| 20 |  |  |
| 21 |  |  |
| 22 |  |  |
| 23 |  |  |
| 24 |  |  |
| 25 |  |  |
| 26 |  |  |
| 27 |  |  |
| 28 |  |  |
| 29 |  |  |
| 30 |  |  |
| 31 |  |  |

Name: ________________________________

Start Date: _____________________________

| **Day** | **Duration in minutes** | **Massage setting** | **Heat setting** |
| --- | --- | --- | --- |
| 1 |  |  |  |
| 2 |  |  |  |
| 3 |  |  |  |
| 4 |  |  |  |
| 5 |  |  |  |
| 6 |  |  |  |
| 7 |  |  |  |
| 8 |  |  |  |
| 9 |  |  |  |
| 10 |  |  |  |
| 11 |  |  |  |
| 12 |  |  |  |
| 13 |  |  |  |
| 14 |  |  |  |
| 15 |  |  |  |
| 16 |  |  |  |
| 17 |  |  |  |
| 18 |  |  |  |
| 19 |  |  |  |
| 20 |  |  |  |
| 21 |  |  |  |
| 22 |  |  |  |
| 23 |  |  |  |
| 24 |  |  |  |
| 25 |  |  |  |
| 26 |  |  |  |
| 27 |  |  |  |
| 28 |  |  |  |
| 29 |  |  |  |
| 30 |  |  |  |
| 31 |  |  |  |
